# 

**Published:** 1816-05

**Authors:** 


CORRESPONDENCE.

Dr. Seeds's Translation of Pariera's Thesis; a Case of sudden Death, with some unusual Symptoms; Review of the Medico-Chirurgical Transactions; and of Dr. Carson on the Circulation, will appear in our next Number.

